# PSCSE: prompt-based contrastive learning with sample filtering for unsupervised sentence embedding

**DOI:** 10.1038/s41598-026-52560-1

**Published:** 2026-05-27

**Authors:** Biao Li, Xuebing Yang, LiPing Xie

**Affiliations:** 1 College of Computer Science and Technology, Taiyuan University of Science and Technology, Peace Street, Taiyuan, 030024 Shanxi China; 2 Artificial Intelligence and Machine Learning Research Team, Institute of Automation, Chinese Academy of Sciences, Zhongguancun Street, Beijing, 100080 China

**Keywords:** Unsupervised sentence representation, BERT, Prompt learning, Computational biology and bioinformatics, Mathematics and computing

## Abstract

Unsupervised sentence representation learning is critical in natural language processing. Recently, contrastive learning methods achieved remarkable performance by optimizing the alignment and uniformity of embedding spaces. Nevertheless, the dominant methods mainly focus on the data augmentation for the positive samples but ignore the sampling approach for the negative samples. Most methods rely on the random in-batch sampling approach for the generation of the negative samples. This approach could result in false negatives and create sampling biases for the model’s discriminative ability. To address this problem, we propose a new method prompt-based contrastive learning with sample filtering for unsupervised sentence embedding (PSCSE). In the proposed model, we used the synthesized hard negatives generated by the “NOT”-style prompts (e.g., “This sentence: [X] does not mean [MASK]”) to optimize the uniformity of the learned representations. In addition, we used the auxiliary encoder for the sample filtering approach to address the false negatives. We conducted experiments on the semantic textual similarity dataset and achieved remarkable performance by surpassing the dominant methods SimCSE, E-SimCSE, and PromptBERT by up to 1.0 points in the average Spearman’s correlation score.

## Introduction

Sentence representation learning^1,2^ plays a crucial role in the area of natural language processing (NLP), where the goal is to obtain high-quality sentence representations that are suitable for different downstream tasks, e.g., semantic similarity calculation and sentence question answering. Especially for resource-poor tasks or computationally expensive domains, unsupervised sentence representation learning has progressively attracted research interest in recent years and has revealed important potential for zero-shot semantic textual similarity^3^, sentiment analysis^4^, and document retrieval^5^. Nevertheless, due to the lack of supervision, the scalability and generalization of sentence representations are limited and the performance of NLP applications remains unsatisfactory.

The advent of large-scale pre-trained language models (PLMs), represented by BERT^6^, Roberta^7^, has benefited many downstream tasks through fine-tuning methods. More recently, in 2024 and 2025, the field has witnessed a paradigm shift towards leveraging Large Language Models (LLMs) and instruction tuning for sentence representation. For instance, recent state-of-the-art models like NV-Embed^8^, BGE-M3^9^, and GritLM^10^ have demonstrated the efficacy of transforming generative models into generalist embedders. However, the original sentence representations obtained by PLMs were found not to be uniformly distributed but to occupy a narrow cone in the vector space, and experiments show that the original BERT performs poorly on semantic similarity datasets, which undoubtedly greatly limits the sentence representation ability of PLMs^11–13^.

To solve this problem, researchers have proposed using contrastive learning to make the sentence representation space as uniform and aligned as possible. Contrastive learning improves the representation space by simultaneously increasing the alignment of positive samples and the uniformity of negative samples. It generally uses various data augmentation methods (e.g. dropout^14^ or token shuffling^15^) to randomly produce various views for every original sentence, and the data-augmented sentence vectors are extremely similar in semantics to the original sentences. After that, input sentences and data-augmented sentences are used as a pair of positive samples, which are pulled together to improve alignment^16^, while input sentences and other sentences are used as negative samples, which are pushed apart to improve the spatial uniformity of sentence representations.

One optimization orientation in contrastive learning is to use more negative samples to encourage the model to learn finer distinctions^17^. However, our experiments show that in the sentence feature learning task, the performance of the model in SimCSE^14^ is best when the batch size is 64 (other sentences in a batch are used as negatives in this work), and as the number of negatives increases, the model performance is degraded instead; this is contrary to what is demonstrated in Tsai et al.^18^. We formalize this phenomenon of degradation as False Negative Collapse. When we choose contrastive negative samples that share semantic similarity with the anchor in the InfoNCE objective function, this creates an incorrect repulsive effect. As we increase the contrastive negative sampling size, the chance of encountering a false negative rises in a monotonic way. Finally, when we optimize the loss function in the face of this false negative problem, we observe that the encoder starts to split semantic neighbors, disrupting semantic continuity and making it difficult to develop compact class clusters (i.e., fragmentation of the data manifold). Also, when we randomly sample negative points, we observe another problem: Boundary Skew. We see that negative points do not uniformly represent the actual data distribution and that we over-optimize the encoder to represent easy negative points. We observe that this creates a distorted representation space that does not have a linear relationship between semantic similarity and cosine distance.

In view of the above problems, we aim to address them using prompt learning^17^ and sample filtering. Hence, we design an unsupervised sentence contrastive learning approach that uses prompts to supplement negative samples, the core of which is to use prompts to make BERT produce more negative samples and to improve the strategy of negative sampling to alleviate the problem of sampling bias. Specifically, we input the randomly sampled negatives into a particular prompt template, and then generate new sentence vectors through BERT. The new sentence vectors are semantically different from the original sentences, and we add these sentence vectors to the negatives to expand the representation space of sentences and make the decision boundary more accurate. In addition, in order to remove false negatives, the method of sample filtering is used to debias, that is, we use an auxiliary model to calculate the sentence similarity between sentences and negative samples, and negative samples with large similarity are filtered out so that false negatives can be removed.

The structure of this paper is as follows: “Prompt-based learning foundations” section represents preliminary work, and our experiment is based on PromptBERT^17^. In “The prompt-based contrastive learning with sample filtering (PSCSE) method” section presents our approach PSCSE. In “Experiments” section introduces our datasets, evaluation methods, and baseline methods, and shows the results of main experiments, ablation experiments, and parametric experiments. Our contributions can be briefly generalized below:

- We propose a method to effectively supplement negatives by using prompts to make BERT generate more effective negatives, making the spatial distribution of sentence representations more uniform.
- We propose sample filtering with the help of auxiliary models to remove false negatives.
- We conducted adequate experiments on seven semantic similarity datasets, and the experimental results showed that both of our proposed innovative methods greatly improve the baseline method, enabling PSCSE to reach a consistently higher and even state-of-the-art average Spearman’s correlation than other popular unsupervised models for sentence representation, such as SimCSE, E-SimCSE, and PromptBERT, for various backbones of pre-trained language models.

## Related work

### Traditional sentence representation methods

The study of sentence representation has progressed under two major paradigms of research: supervised and unsupervised learning, both of which constitute basic elements of semantic modeling. Related work explores the effect of various architectures and tasks on the separation, generalization capability, and semantic accuracy of sentence embeddings, ranging from the early work on Siamese networks to the use of transformer language models. The importance of the overview is that it outlines the differences between the methodologies used to obtain the semantic representation of a sentence and explains the way these differences influence the geometry of the embeddings.

Sentence representation learning^1,2^ is extremely critical for various NLP downstream tasks. Previous studies can be broadly classified into two classes: supervised methods and unsupervised methods. Among supervised methods, Conneau^19^ proposed a labeled SNLI dataset^20^ suitable for training sentence embeddings. InferSent^19^ used SNLI to train a Siamese BiLSTM neural network to learn sentence representations. Yang^21^ proposed using Siamese-DAN and Siamese transformer architectures to train on conversations from Reddit, and achieved good results on the STS-Benchmark dataset. Sentence-BERT^22^ uses Siamese/triplet neural networks to fine-tune BERT on the NLI dataset to make the sentence embeddings generated by BERT better for comparative similarity, and achieves good results on seven semantic similarity datasets.

In contrast to supervised approaches, unsupervised methods focus on learning representations without explicit label guidance. Skip-thought^1^ trains an encoder–decoder architecture to predict surrounding sentences. Universal Sentence Encoder^23^ uses the SNLI dataset^20^ to train a transformer network to learn to reconstruct surrounding sentences in paragraphs. After the pre-trained model BERT was proposed, some works linearly transform the sentence representation space obtained by BERT, e.g. BERT-flow^13^, and BERT-whitening^24^, to solve the problem of sentence representation space anisotropy. It is worth noting that encoder-only architectures continue to evolve, with recent works like ModernBERT^25^ extending the context length and efficiency of BERT-style models for retrieval tasks. There are also recent works^14,15,26^ using contrastive learning to address the same problem; this contrast-based approach allows for a better sentence representation space^16^.

### Contrastive learning for sentence representation

Contrastive learning has proven to be an important approach to discriminative embedding learning. It is based on the theoretical principle of simultaneously maximizing alignment and uniformity. Semantic augmentation techniques, as well as encoders using the momentum approach to adapt the paradigm to natural language processing data, have been employed in the application of contrastive learning in natural language processing. Furthermore, this subsection presents the various methodologies in a unified conceptual approach.

The purpose of contrastive learning is to learn a uniform embedding space by pulling representations with small distances together and pushing representations with large distances apart. Originally used in the area of computer vision^27^, contrastive learning usually first performs data augmentation (e.g. cropping and resizing^28^) on the original image to generate a pair of semantically similar positive samples, and treats other samples in a batch as negatives. Additionally, the application of structured priors or contrastive approaches to improve the quality of the features has been demonstrated in various other related fields. For instance, recent research in the fields of image deblurring techniques^29^, such as CGD-TraP^30^, multi-modal fusion techniques, such as DDMSNet^31^, as well as robust feature representation techniques such as VSPBFR^32^, has adopted prior-driven approaches. These approaches have the same basic principle as the ones adopted in the present research: the application of constraints or structured priors in the form of visual or linguistic information to greatly improve the discriminative power. In sentence representation learning, a pair of positive examples are obtained by performing different data augmentation operations on sentences , e.g. token shuffling^15^, dropout^14^ and back translation^26^. This gives the model better representation space separability.

In addition to data augmentation, some works focus on improving the negative sampling strategy in contrastive learning. Recent studies in 2024 have further refined this paradigm by incorporating AI feedback to select higher-quality negatives^33^. Moco^34^, E-SimCSE^35^ use a momentum contrast encoder, which sustains several prior mini-batches’ model outputs in a queue, to expand negatives. Major approaches today highlight positive augmentation to the detriment of fully exploring negative sampling mechanisms. Traditional approaches to hard negative mining attempt to obtain hard negatives from the dataset; however, this is done at a high computational cost and with many false negatives. In contrast, PSCSE uses a generative model. Rather than trying to obtain hard negatives, we generate samples through “NOT”-style prompts. This is because these samples are lexically similar yet syntactically different. Furthermore, unlike other probabilistic approaches to debiasing that rely on implicitly reducing false negatives through distribution estimation, we have explicitly used a sample filtering approach through an additional encoder. This is because hard sampling is a safeguard against semantic collision within our unsupervised model.

### Prompt-based learning in NLP

The prompt-based learning paradigm involves the incorporation of prior knowledge into a pre-trained language model by inserting a sentence into a structured context through a template-driven representation, effectively reformulating the representation of a sentence’s meaning in a cloze prediction task setting. The major factor in the successful execution of the prompt-based learning paradigm is the quality of the templates, as the discussion in the subsequent section reveals regarding the integration of positive templates from both manual and automated template development methods.

Different from the previous “Pre-train, Fine-tune” paradigm, prompt-based learning creates a new NLP paradigm, “Pre-train, Prompt, Predict”. Prompt-based learning is based on language models that directly simulate text probabilities and is well suited for low-resource scenarios. Its core is to convert the input *X* into a textual string prompt $X^{\prime}$ through the prompt template, where there are some unfilled slots, and then to use the language model to perform probability filling on the unfilled information to obtain the final sequence $\hat{X}$, from which the final output *Y* can be obtained. The main work of prompt-based learning lies in the construction of prompt templates. The construction methods are divided into manual construction^36,37^ and automatic construction^38–40^.

## Prompt-based learning foundations

For positive example construction, we follow the PromptBERT^17^ approach to augment the input samples. For each input sentence, let *X*, and $X^{+}$ represent the original sentence and the positive sample sentence, respectively. Here *X* and $X^{+}$ have the same textual content but use different prompts, treating other sentences $X_{j}$ in a batch as negative samples. We encode them with a masked language model (MLM) under different prompts:

1 $$\begin{aligned} \begin{aligned}&\mathrm{input:}\;\;\;\;\;\;\text{This sentence: }``X\text{'' means [MASK].}\\&\mathrm{positive:}\;\;\;\text{This sentence of} \; ``X^{+}\text{'' means [MASK].}\\ \end{aligned} \end{aligned}$$

We used a manually constructed template, passing the input *X* through two similar prompt templates to output two sentence vectors, and then use the MLM e.g. BERT^6^ and RoBERTa^7^ to perform probability filling on the “[MASK]”. At the same time, the two templates in Eq. (1) are highly similar, which is equivalent to adding a noise to the input, essentially performing data augmentation, and treat them as a pair of positive examples $(X, X^{+})$.

At the same time, we treat the hidden state of the token at the masking $h_{[mask]}$ as the embedding h of the sentence. In training, we add a multilayer perceptron layer with nonlinear activation function on $h_{[mask]}$ to acquire h:

2 $$\begin{aligned} h=\operatorname {Tanh}\left( MLP\left( h_{[mask]}\right) \right) . \end{aligned}$$

Use InfoNCE loss to pull the embeddings of positive pairs together, and push those of negative pairs apart:

3 $$\begin{aligned} \mathcal {L}_{\text{InfoNCE }}=-\log \frac{e^{ \operatorname {sim}\left( h_{i}, h_{i}^{+}\right) / \tau }}{\sum _{j=1}^{N} e^{ \operatorname {sim}\left( h_{i}, h_{j}^{-}\right) / \tau } }. \end{aligned}$$

where *sim* means cosine similarity $\frac{\mathbf{a}^{\top } \mathbf{b}}{\left\| \mathbf{a}\right\| \cdot \left\| \mathbf{b}\right\| }$, *τ* means temperature coefficient, where N means batch size. $h_{i}^{+}$ represents the sentence representation of $h_{i}$ after data augment, $h_{j}^{-}$ means the sentence representation of other samples in the mini batch , here as negative samples in the contrastive loss. With this loss, the distance between pairs of positive examples is narrowed, and the distance between pairs of negative examples is widened.

## The prompt-based contrastive learning with sample filtering (PSCSE) method

**Fig. 1:**
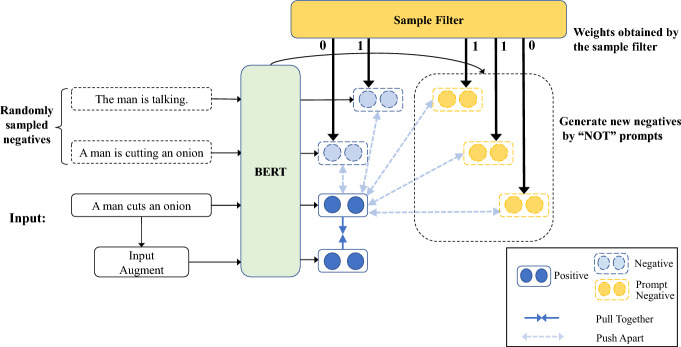
The overview of our approach PSCSE with prompt-based negatives and sample filter.

Our proposed method focuses on increasing the uniformity and separability of the sentence representation space using prompts. In this approach, we design a novel way of augmenting negatives, and the newly generated negative samples are semantically different from the previously sampled negatives. Before the samples are input into the model, false negatives are removed by a sample filtering method to reduce the bias caused by random sampling. An overview of our model is shown in Fig. 1. In the following, we will detail the two methods of generating prompt-based negatives and sample filtering.

### Generating prompt-based negatives

Firstly, in addition to using negatives in a batch, all original samples are transformed into sentence vectors by adding “NOT” prompt templates (Eq. 4). Before entering the sentence into the BERT encoder, we first enter the sentence into the prompt template we designed, that is, we replace *X* in Eq. (4) with the original input sentence and make BERT restore the token at [MASK]. After passing through the BERT encoder, we use the embedding of [MASK] as the embedding of the sentence (Eq. 2), thus generating a new negative sample. In the process of contrastive learning, the original negative sample and the newly generated negative sample are added to the contrastive loss function at the same time.

4 $$\begin{aligned} \begin{aligned} \text{This sentence:}\;\; ``X \text{'' does not mean [MASK].}\\ \end{aligned} \end{aligned}$$

Through this “NOT” prompt template, the negative samples are effectively supplemented. To be specific, this deliberate prompt design ensures two critical properties that produce meaningful semantic differences and effective training signals for contrastive learning:

- Semantic Contradiction: By introducing the semantic condition “does not mean”, the model is forced to distinguish between the original intent and its logical opposite. This acts as a directional perturbation, projecting the generated representation away from the anchor’s semantic centroid.
- Lexical Overlap (Hardness): Since the prompt retains the entire original sentence *X*, the self-attention mechanism aggregates the original lexical features. This ensures the synthesized negative sample remains extremely close to the anchor in the lexical space. Functioning as a “synthesized hard negative”, it effectively prevents the model from relying on simple word-matching shortcuts during optimization.

To strictly control the risk of the prompt generating mere paraphrases or unstructured noise, our Sample Filter (“Filtering false negatives” section) serves as an intrinsic diagnostic tool: it quantifies the semantic drift by calculating the cosine similarity. Generated samples that fail to diverge sufficiently (i.e., retain high similarity to the anchor) are explicitly identified as false negatives and discarded.

As prompted, we improved the original InfoNCE loss function (Eq. 3) as follows:

5 $$\begin{aligned} L=-\log \frac{e^{\operatorname {sim}\left( h_{i}, h_{i}^{+}\right) / \tau }}{\sum _{h^{-} \in \{h_{j}\} \cup \{\hat{h}_{i}\} \cup \{\hat{h}_{j}^{-}\}} e^{\operatorname {sim}\left( h_{i}, h^{-}\right) / \tau }} . \end{aligned}$$

where $\hat{h}_{i}$, $\hat{h}_{j}^{-}$ represent the new negatives generated by prompts, which is obtained by putting $X, X_{j}$ enter Eq. (4) and probability filling on the “[MASK]”. With the prompt template with “NOT”, the semantics of *X* and $X_{j}$ are changed, and the negative representation space is expanded. We further acknowledge that the process of generating samples using the provided prompts relies on the model’s understanding of negation statements that might occasionally go wrong and result in generated samples that remain semantically similar to the anchor (false negatives). Therefore, the Sample Filtering mechanism (as discussed in “Filtering false negatives” section) acts as an important quality control step that helps prevent the risk of false negatives by evaluating the semantic similarities between the generated samples and the anchor, thus maintaining the quality of the contrastive learning objective.

As shown in Fig. 1, the dark blue samples represent a pair of positive samples, which are generated by prompts. The light blue samples represent negative samples that are randomly sampled in a batch. The yellow sample represents the new negative sample generated by us using the prompt, that is, the original negative sample is input into the prompt template we designed, and through this special prompt template, the generated negative sample is semantically different from the original negative sample, so as to achieve the purpose of filling the sentence feature space as much as possible. Returning to the original discussion of the intuition behind the proposed method, and moving forward into the proposed scheme itself, it appears that the objective of the Sample Filter is to ensure that similar semantic meanings are not pushed apart within the semantic embeddings. This is achieved through the Sample Filter assigning a weight to each candidate negative sample, such that false negatives are suppressed if there is a semantic similarity with the reference sentence, and true negatives are kept if there is low semantic similarity, thus ensuring that the semantic embeddings are not pushed apart if they are similar.

### Filtering false negatives

Since the negative sample space is expanded, false negatives that have similar semantics to the input are more likely to exist during contrastive learning. Therefore, a sample filtering method is adopted to remove false negatives. In this paper, “false negatives” are defined as negative samples whose semantics are highly similar to the anchor sentence, among which the extreme cases are those where the negative sentence is actually a paraphrase or even an equivalent expression of the anchor or positive sample. When such samples are treated as negatives in contrastive learning, the loss still pushes them away from the anchor, which compresses semantic clusters that should stay close and yields a fragmented structure of the representation space. Thus, sample filtering is used to reduce the destructive effect of these samples on the sentence representation space.

In this section, we propose a sample filtering method to remove false negatives. The cosine similarity between a candidate negative sentence and the anchor sentence is calculated using the unsupervised auxiliary model to detect false negatives for the sample filter. If the result is above the threshold level of *β*, the sample is declared a false negative and not used for computing the loss; otherwise, it is a true negative.

Specifically, we add an unsupervised auxiliary model to pick out false negatives from negatives, and false negatives do not participate in the contrastive loss computation. Given a negative sample, where $h^{-}$ comes from $h_{j}$, $\hat{h}_{i}$ or $\hat{h}_{j}^{-}$, due to the lack of real labels or semantic similarity in unsupervised sentence representation learning, we choose to use an auxiliary model to calculate the semantic similarity between $\hat{h}_{i}$ and $h^{-}$, and the weight $\omega _{h^{-}}$ is determined by the semantic similarity as :

6 $$\begin{aligned} \omega _{h^{-}}=\left\{ \begin{array}{ll} 0,& \quad \operatorname {sim}_{a}\left( h_{i}, h^{-}\right) \ge \beta \\ 1,& \quad \operatorname {sim}_{a}\left( h_{i}, h^{-}\right) <\beta \end{array}\right. \end{aligned}$$

where *β* is a hyperparameter of negative weight threshold, $\operatorname {sim}_{a}( h_{i}, h^{-})$ represents the cosine similarity score calculated by the auxiliary model, and $\omega _{h^{-}}$ means the weight of negative. In this way, negatives with high semantic similarity to the original input sentence representation will be considered as false negatives, and their weight is set to 0. Based on this weight, we optimize the sentence representation contrastive loss function as Eq. (7). In addition, in the selection of auxiliary models, we choose the unsupervised SimCSE model.

7 $$\begin{aligned} L=-\log \frac{e^{\operatorname {sim}\left( h_{i}, h_{i}^{+}\right) / \tau }}{\sum _{h^{-} \in \{{h}_{j}\} \cup \{\hat{h}_{i}\} \cup \{\hat{h}_{j}^{-}\}} \omega _{h^{-}} \times e^{\operatorname {sim}\left( h_{i}, h^{-}\right) / \tau }} . \end{aligned}$$

As shown in Fig. 1, before the sentence enters the BERT model, we use the auxiliary model to calculate the direct similarity of the sentence. If it is greater than a certain threshold, it is judged to be a false negative, and this auxiliary model is the Sample Filter. In the randomly sampled negatives, the sentence “A man is cutting an onion” is semantically similar to the input sentence “A man cuts an onion”, so it is clearly an inappropriate negative sample. In this case, the sampled negative example is what was previously referred to as a false negative. Our filter calculates the similarity between the two sentences using an auxiliary model. If the similarity is greater than a threshold *β*, the filter sets the weight of the false negative to 0, preventing it from being included in the loss calculation.

### Integrated training algorithm

To provide a unified view of the proposed framework, we summarize the complete training pipeline in Algorithm 1. The process begins with constructing positive pairs using prompt templates. Subsequently, for each anchor in the batch, we synthesize additional hard negatives using the “NOT” prompt. These candidates, along with the standard in-batch negatives, undergo a validity check via the Sample Filter, which assigns binary weights $(\omega _n)$ to exclude false negatives. Finally, the model is optimized using the re-weighted InfoNCE loss (Eq. 7). This integrated procedure ensures that the encoder learns from a diverse and semantically refined set of negatives while maintaining computational feasibility.

**Algorithm 1:**
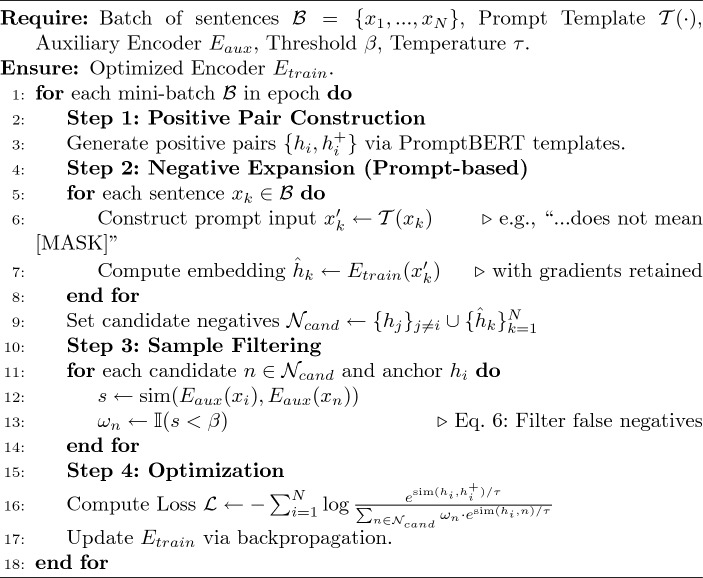
PSCSE training pipeline

## Experiments

### Dataset

Following existing works^14,15^, we first use unsupervised training to obtain our model and then evaluate it on supervised STS tasks. The STS task consists of seven subtasks: STS12–16, STS-B, and SICK-R^41–47^. Each STS subtask consists of multiple sentence pairs, each annotated with a semantic similarity score ranging from 0 to 5. A higher score indicates greater semantic similarity between the sentences. Table 1 summarizes the details of these subtasks.

**Table 1:** Statistic information of STS task.

Task	STS12	STS13	STS14	STS15	STS16	STS-B	SICK-R
Train	0	0	0	0	0	5749	4500
Dev	0	0	0	0	0	1500	500
Test	3108	1500	3750	3000	1186	1379	4927

### Evaluation metric

During model evaluation, we use our model to compute the cosine similarity of each sentence pair, using only the test set for evaluation. For a pair of sentences, the semantic similarity for the STS task ranges from 0 to 5, whereas the cosine similarity ranges from − 1 to 1. To account for this range difference, we use a rank-based Spearman’s correlation coefficient.

In detail, for a subtask with n sentence pairs, we rank the similarity scores to obtain the score rank $R_{G}$ for each sentence pair. We then use our model to compute and rank the cosine similarity to get the estimated rank RE $R_{E}$ for each pair. Then the rank-based Spearman’s correlation coefficient *ρ* of the STS task is defined as:

8 $$\begin{aligned} \rho =1-\frac{6\sum _{i=1}^{n}\left( R_{E}^{i}-R_{G}^{i}\right) ^{2}}{n\left( n^{2}-1\right) } \end{aligned}$$

The range of *ρ* above is − 1 to 1, and the higher the value of *ρ*, the better the sentence representation of the model and the stronger the semantic understanding ability. We calculate the Spearman’s correlation coefficient *ρ* for each subtask separately, and then average it as a metric to evaluate the performance of the model.

### Experiment setup

Our method is implemented on the open-source Python machine learning libraries PyTorch 1.7.1 and HuggingFace Transformers.^1^ We randomly sample a million sentences from Wikipedia as the unsupervised training corpus (this corpus is unlabeled). During training, we perform our experiments on Nvidia RTX 3090 devices. In the implementation details, we train the model for only one epoch and set the dropout rate to 0.1, the temperature coefficient T = 0.05, the threshold *β* of the sample filter to 0.9, and use the Adam optimizer^48^. When we use BERT-base-uncased and RoBERTa-base as the base models of our approach, we set the batch size to 216 and the learning rate to 1e−5. When we use BERT-large-uncased and RoBERTa-large as the base models, we set the batch size to 64 and the learning rate to 5e−6 to avoid memory overflow problems. For prompt-based negative samples, we try different templates to test the performance of the model, and finally choose the template “This sentence: “*X*” does not mean [MASK].”. With regard to each batch, we generate n *×* batch size prompt-based negative samples and add them to the original negative samples, where n is 1, 2, 1, 2 for BERT-base-uncased, BERT-large, RoBERTa-base-uncased, and RoBERTa-large, respectively. We evaluate our model every 125 steps on the validation set of STS-B and SICK-R, and retain the best checkpoints.

In terms of computational complexity, there is additional cost associated with our proposed model compared to normal SimCSE because of the prompt-based negative generation and the use of the sample filter. In this regard, there is a forward pass of an auxiliary model to obtain cosine similarities. However, this additional cost is limited to the training phase. In terms of inference, PSCSE is equivalent to a normal BERT model. Furthermore, we note that this additional cost is limited to one epoch, and because this model is highly parallelized, this additional cost is justified with respect to our performance gains of up to 1.0 points on downstream tasks.

### Baselines

We compare our method with existing unsupervised sentence representation learning methods, including methods without and with BERT as the backbone

1. *GloVe*^49^ is a word representation tool based on global word frequency statistics. These vectors capture some semantic features between words, such as similarity and analogy. The semantic similarity between two words can be calculated by vector operations, such as Euclidean distance or cosine similarity.
2. *USE*^23^ uses a Transformer encoder and is trained in an unsupervised manner using the Skip-Thought^1^ method. USE obtains the current sentence vector through the encoder to predict the words in the sentences before and after the current sentence, and the representation of the sentence is obtained by averaging the word representations at each position output by the encoder.
3. *Whitening*^24^ uses a whitening operation to refine representations and reduce dimensionality. That is, the mean of BERT’s output vector matrix is converted to 0, and its covariance matrix is converted to a unit matrix.
4. *Flow*^13^ transforms the BERT encoder’s sentence representations into an isotropic and evenly distributed space. The standard Gaussian distribution happens to be an isotropic space and has a convex density function, and the semantic distribution is smoother and more uniform.
5. *Cert*^26^ uses back translation to create an augmentation of the original sentence. It then fine-tunes a pre-trained language encoder by predicting whether two augmented sentences are from the same sentence, to enhance sentence representations.
6. *ConSERT*^15^ investigates various text enhancement strategies for contrastive learning of sentence embedding, *e.g.* adversarial attack, token shuffling, cutoff, dropout.
7. *SimCSE*^14^ designs a simple contrastive learning approach that uses dropout for data augmentation, and this simple data augmentation method is more effective for sentence representation than several methods used by ConSERT.
8. *E-SimCSE*^35^ uses momentum contrast to expand the number of negative pairs without additional computation. The data are augmented by word repetition for the input sentences, and then the two sentences are used as a pair of positive examples.
9. *PromptBERT*^17^ proposes inputting a sentence into two similar prompt templates as a pair of positive examples in contrastive learning to learn sentence representations.
10. *SNCSE*^50^ improves unsupervised sentence embeddings by introducing negated “soft negative samples” and a Bidirectional Margin Loss (BML) to effectively decouple textual similarity from semantic similarity.
11. *NV-Embed*^8^ A recent state-of-the-art embedding model based on a massive 7B-parameter Large Language Model (Mistral-7B). We include its unsupervised/first-stage performance to represent the current upper bound of large-scale representation learning.
12. *ModernBERT*^25^ Released in late 2024, this model represents the latest modernized encoder architecture, pre-trained on up to 2 trillion tokens with structural optimizations like Rotary Positional Embeddings (RoPE). We compare its base version to evaluate our prompt strategy against the latest architectural advancements.

### Main results

In order to validate the effectiveness of our proposed model, we use BERT-base-uncased, BERT-large-uncased, RoBERTa-base, and RoBERTa-large as our base models. Table 2 shows the performance of our method and baseline methods on seven STS tasks.

From the results in the Table 2, we can observe that the sentence representations of native PLMs are the worst, primarily because directly using native PLMs to represent sentences is susceptible to the anisotropy issue. Sentence representations of non-BERT methods outperform native BERT, and the performance of USE is higher than that of GloVe. This is because USE uses a Transformer encoder to encode sentences, which also shows the power of the Transformer model.

For other BERT-based models, we can find that the performance of BERT-flow^13^ and BERT-whitening^24^ is far better than that of native BERT. This is because they add flow transformation and whitening operations to the BERT pre-trained model, respectively, to solve the problem of vector anisotropy and the non-uniform distribution of native BERT sentence embeddings. Secondly, methods based on contrastive learning to represent sentences outperform other baseline methods in most cases. The reason is that contrastive learning can further improve the uniformity and alignment of the sentence embedding space, which leads to better separability of sentence representations. Furthermore, among these methods using contrastive learning, PromptBERT^17^ performs the best, suggesting that using a prompt template is a more effective method for positive sample data augmentation.

To further assess the impact of negative sample optimization, we extend the comparison beyond SNCSE^50^ to include stronger and more recent baselines such as NV-Embed^8^ and ModernBERT-based^25^ representations. SNCSE, a concurrent state-of-the-art approach, leverages soft-negative estimation and achieves strong performance (78.97 on BERT-base). As shown in Table 2, PSCSE attains a slightly higher average score (79.10), indicating that our method remains competitive with, and in some cases marginally surpasses, this advanced soft-labeling strategy.

**Table 2:** The table evaluates the sentence embedding performance of our model for seven STS tasks, and we chose to use the Spearman’s correlation as the evaluation metric.

	Models	STS12	STS13	STS14	STS15	STS16	STS-B	SICK-R	Avg.
Non-BERT	Glove(avg.)^†^	55.14	70.66	59.73	68.25	63.66	58.02	53.76	61.32
	USE^†^	64.49	67.80	64.61	76.83	73.18	74.92	76.69	71.22
BERT-base	BERT-flow^‡^	58.40	67.10	60.85	75.16	71.22	68.66	64.47	66.55
	BERT-whitening^‡^	57.83	66.90	60.90	75.08	71.31	68.24	63.73	66.28
	Cert^†^	54.26	64.03	54.28	68.19	67.50	63.27	66.91	62.63
	ConSERT^#^	64.64	78.49	69.07	79.72	75.95	73.97	67.31	72.74
	SimCSE^‡^	68.40	82.41	74.38	80.91	78.56	76.85	72.23	76.25
	E-SimCSE^#^	**73.40**	83.27	77.25	82.66	78.81	80.17	72.30	78.27
	PromptBERT^$^	71.56	84.58	76.98	84.47	80.60	81.60	69.87	78.54
	SNCSE	70.67	**84.79**	76.99	83.69	80.51	81.35	**74.77**	78.97
	PSCSE(Ours)	72.17	84.23	**77.39**	**84.86**	**80.99**	**82.13**	71.91	**79.10**
BERT-large	BERT-flow^‡^	62.82	71.24	65.39	78.98	73.23	72.72	63.77	70.07
	BERT-whitening	64.34	74.60	69.64	74.68	75.90	72.48	60.80	70.35
	Cert^†^	52.04	62.59	54.25	71.07	66.71	63.84	66.53	62.43
	ConSERT^#^	70.69	82.96	74.13	82.78	76.66	77.53	70.37	76.45
	SimCSE^‡^	70.88	84.16	76.43	84.50	79.76	79.26	73.88	78.41
	E-SimCSE^#^	73.21	85.37	77.73	84.30	78.92	80.73	74.89	79.31
	PromptBERT	**73.21**	86.42	77.99	85.09	80.14	81.32	72.76	79.56
	SNCSE	71.94	**86.66**	78.84	85.74	80.72	82.29	**75.11**	80.19
	PSCSE(Ours)	72.90	86.13	**79.45**	**85.94**	**81.04**	**82.92**	73.42	**80.25**
RoBERTa-base	BERT-whitening^‡^	46.99	63.24	57.23	71.36	68.99	61.36	62.91	61.73
	Cert^†^	62.34	78.60	68.65	79.31	77.49	79.93	71.97	74.04
	SimCSE^‡^	70.16	81.77	73.24	81.36	80.65	80.22	68.56	76.57
	E-SimCSE^#^	69.90	82.50	74.68	83.19	80.30	80.99	70.54	77.44
	PromptBERT^$^	73.94	84.74	77.28	84.99	81.74	81.88	69.50	79.15
	SNCSE	70.62	84.42	77.24	84.85	81.49	**83.07**	**72.92**	79.23
	PSCSE(Ours)	**73.99**	**85.29**	**77.86**	**85.49**	**82.10**	82.95	72.58	**80.04**
RoBERTa-large	BERT-whitening	64.17	73.92	71.06	76.40	74.87	71.68	58.49	70.08
	Cert^†^	57.60	72.14	62.25	71.49	71.75	77.05	67.83	68.59
	SimCSE^‡^	72.86	83.99	75.62	84.77	81.80	81.98	71.26	78.90
	E-SimCSE^#^	73.20	84.93	76.88	84.86	81.21	82.79	72.27	79.45
	PromptBERT	73.31	84.81	77.48	84.35	81.93	82.47	71.98	79.47
	SNCSE	73.71	**86.73**	80.35	**86.80**	83.06	84.31	**77.43**	**81.77**
	PSCSE(Ours)	**75.32**	85.92	**80.46**	86.27	**83.54**	**84.57**	74.93	81.57
Mistral-7B	NV-Embed	76.22	86.3	82.09	87.24	84.77	86.14	82.8	83.65
ModernBERT	ModernBERT-base	70.45	85.12	78.60	86.10	81.55	81.28	70.62	79.10

The best performing method is shown in bold font. ^†^Results from^51^, ^‡^Results from^14^, ^#^Results from^35^, ^$^Results from^17^, NV-Embed results are from^8^, and all other results are replicated or reevaluated by ourselves.

In contrast to SNCSE, which relies on numerically derived soft labels and additional estimation overhead, PSCSE adopts a more direct “Generate-and-Filter” mechanism. By explicitly constructing hard negatives through textual prompts and subsequently filtering unreliable samples, PSCSE avoids the complexity of continuous similarity calibration while still achieving comparable or better performance. This suggests that structurally guided hard-negative synthesis can serve as an effective alternative to soft-negative modeling.

When scaling to stronger encoders, a more nuanced trend emerges. On RoBERTa-large, SNCSE achieves the best overall performance (81.77), while PSCSE obtains a closely matched score (81.38). Notably, PSCSE still outperforms SNCSE on several individual STS benchmarks, indicating that its advantages are task-dependent rather than uniformly distributed. This suggests that the proposed method captures complementary aspects of semantic similarity, even when it does not yield the highest average score.

Furthermore, compared with massive embedding models such as the 7B-parameter NV-Embed (83.65) and newer architectures like ModernBERT-base (79.10), PSCSE remains highly competitive despite operating under a significantly more lightweight (110M parameters) and prompt-driven framework. This highlights that the performance gains of PSCSE are not solely dependent on massive model scale or extensive 2T-token pretraining, but rather stem from more effective optimization of the negative sample space.

Finally, PSCSE demonstrates consistent improvements over prompt-based baselines such as PromptBERT. Since these methods share the same augmentation backbone, the observed gains can be attributed primarily to the proposed negative sampling and filtering strategy. Empirically, PSCSE yields stable improvements of approximately + 0.5 to + 1.0 points across different encoder sizes (BERT/RoBERTa, Base/Large), confirming that refining the negative sample space plays a critical role independent of the underlying encoder capacity.

Statistical significance and robustness analysis. To check if these improvements in performance are statistically sound and not due to random selection of seeds during training, a statistical significance analysis was performed. The experiments were repeated for PSCSE and the top-performing baseline (PromptBERT) model using a different random seed each time (42, 123, 2025) to check their performance on a BERT-base-uncased backbone model. As shown in Table 3, PSCSE shows high stability in its performance since it achieves an average of 79.09 ± 0.14 in Spearman’s correlation while maintaining higher performance than PromptBERT (78.53 ± 0.14) in all experiments.

**Table 3:** Robustness analysis on BERT-base over 3 random seeds.

Model	Seed 1	Seed 2	Seed 3	Avg. Spearman (*μ* ± *σ*)
PromptBERT	78.54	78.39	78.66	78.53 ± 0.14
PSCSE (Ours)	79.10	78.95	79.22	79.09 ± **0.14**

We report Mean ± Standard deviation.

### Ablation study

Our proposed PSCSE uses prompts to generate new negative samples, and we add a sample filter to remove false negatives. To further validate their effectiveness, we perform ablation studies on these two constituents on seven STS tasks and show the Spearman’s correlation coefficients of the BERT-base model on STS-B and SICK-R, as well as the mean Spearman’s correlation coefficient for the seven STS tasks.

As shown in Table 4, the addition of each component improves the performance of the method, and the combination of the two components improves the performance even more. This suggests that both prompt-based negative sample augmentation and sample filtering are important. In addition, the performance loss of the model with the sample filter removed is larger, which is caused by the existence of false negatives.

Regarding the accuracy of false negative detection, we acknowledge that in an unsupervised setting without ground-truth labels, it is difficult to quantify the precise precision and recall of the filter. However, the consistent performance gain (+ 0.51 on average) provided by the filter suggests that the cosine-similarity-based heuristic (threshold *β =0.9*) effectively aligns with semantic equivalence. By filtering out samples with extremely high similarity, the module acts as a coarse-but-effective safety mechanism, trading off a small number of true negatives for the elimination of destructive false negatives.

**Table 4:** Ablation studies of PSCSE on 7 STS tasks.

Task	STS-B	SICK-R	STS-Avg.
Baseline	81.60	69.87	78.54
+Prompt-based negative	81.41	71.11	78.59
+Sample filter	81.88	70.91	78.88
+PSCSE(Ours)	82.13	71.91	79.10

### Parameter analysis

For parameter analysis, we investigate the effect of the multiplier n for negative sample augmentation and the threshold *β* of the sample filter. Here, n denotes the number of prompt-based negative samples generated for each original negative sample, and *β* is the threshold for filtering false negatives. Negative samples with a similarity higher than *β* are filtered out.

#### Multiples of negative

**Fig. 2:**
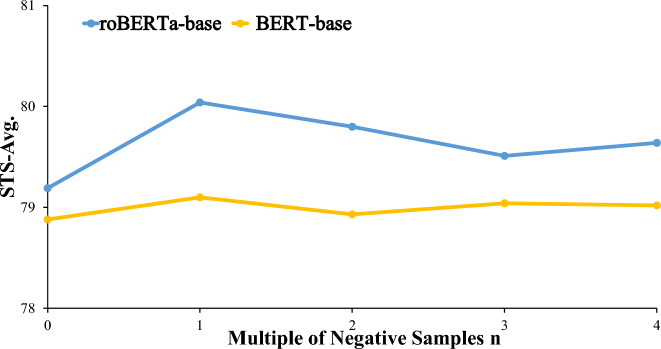
Performance tuning w.r.t multiple of negative samples.

Our goal is to increase the uniformity of the sentence representation space by expanding negative samples through prompts, so the number of negative samples will greatly affect the representation of sentences. Figure 2 shows the influence of negative sample expansion multiples on sentence representation. BERT-base-uncased and RoBERTa-base models were used as base models to select different negative sample expansion multiples for fine-tuning, and the average Spearman’s correlation coefficient on the seven STS tasks was used as the evaluation index. As shown in Fig. 2, for the BERT-base-uncased and RoBERTa-base models, the performance of the model is best when n = 1. This is because if *n* is too large, there will be more false negatives affecting the representation of the sentence. The larger the pool of negative examples, the more likely the model is to include semantically similar examples to the anchor in the negative set, thus disturbing the local structure of the embeddings because the model is forced to push apart examples that should be similar.

It is important to note that this observation regarding *n=1* is specific to the Base-sized models. For Large models (BERT-large, RoBERTa-large), we found in our main experiments that a higher multiple (*n=2*) yields better performance. We attribute this to the higher model capacity of Large architectures, which allows them to leverage a more diverse and extensive set of negative samples for refining decision boundaries without being overwhelmed by the potential noise.

#### Filter threshold

**Fig. 3:**
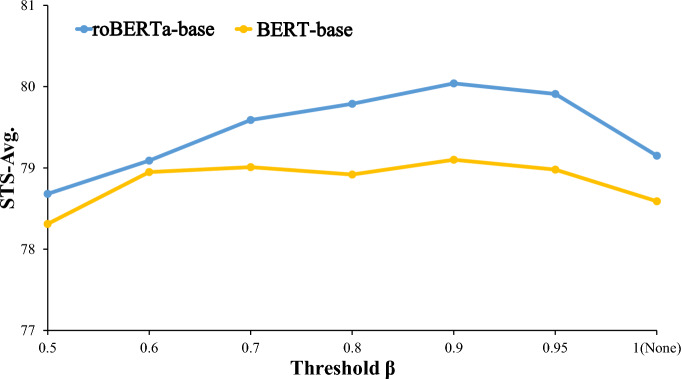
Performance tuning w.r.t threshold *β*.

Figure 3 shows the influence of the threshold *β* of the sample filter on sentence representation. We use BERT-base-uncased and RoBERTa-base as the base models in our approach, and choose different filter thresholds *β* for experiments. Negative samples with similarity higher than the threshold *β* are filtered out. We use the average Spearman’s correlation coefficient of the model on the seven STS tasks to represent the performance of the model. Experiments show that the sentence representation effect of the model is best when the threshold *β* is set to 0.9, and if *β* is too large or too small, the sentence representation ability of the model is reduced. This is because if *β* is too small, more negative samples will be filtered out, and non-false-negative samples will be incorrectly determined as false negatives, so that the model cannot recognize sentences with similar semantics. A threshold that is too small compresses the range of similar scores and makes the model unable to numerically differentiate between true negatives and false negatives. Conversely, if *β* is too large, false negatives cannot be well filtered out, because a threshold that is too large increases the acceptance region of the negative samples and retains similar semantic sentences within the negative set, thus hampering the capability of the model to impose semantic boundaries during the contrastive training procedure.

Notably, while the choice of *β =0.9* provides the best performance for the STS task in the present experiments, this fixed choice of threshold might not necessarily be optimal for all domains. We further observe that the best *β* might depend on the data and the density of the semantics within the corpus used for the task. For future work, we aim to explore the option of using an adaptive approach for choosing the best *β* by estimating the distribution of the similarities within the batch of samples and using that to select the best choice for *β* (e.g., by choosing the top-k percentiles).

### Computational cost analysis

As discussed in “Filtering false negatives” section, PSCSE introduces an auxiliary encoder for the Sample Filter. To provide a transparent view of the computational overhead, we compare the training time and memory usage of PSCSE with the standard SimCSE^14^ in Table 5 (evaluated on a single NVIDIA RTX 3090 GPU with a batch size of 64).

As expected, the dual-encoder mechanism increases the training memory from 7.5 GB to approximately 14.8 GB, and extends the training time per epoch from 12 minutes to 22 minutes. However, we argue that this overhead is highly acceptable for two critical reasons:

- *Manageable training requirements*: A memory footprint of 14.8 GB easily fits within the capacity of modern consumer-grade GPUs (e.g., RTX 3090 or A5000), meaning that PSCSE does not require prohibitive or specialized hardware clusters to train.
- *Zero inference overhead*: Most importantly, the auxiliary filter encoder is strictly a training-time component. Once the model is trained, the filter is discarded. During inference, PSCSE utilizes only the primary encoder, resulting in an inference time (*∼* 15 ms per batch) that is mathematically and empirically identical to SimCSE.

Given the substantial performance gain (+ 2.85 points over SimCSE) without any sacrifice to real-world deployment speed, the one-time training overhead introduced by our ‘Generate-and-Filter’ paradigm is a highly worthwhile trade-off.

**Table 5:** Comparison of computational efficiency on a single RTX 3090 GPU (Train Time: per epoch; Infer Time: per batch; Batch size = 64).

Method	Memory (GB)	Train time (min)	Infer time (ms)	Avg.
SimCSE (BERT-base)	~ 7.5	~ 12	~ 15	76.25
PSCSE (BERT-base)	~ 14.8	~ 22	~ 15	79.10

## Conclusion

In this paper, we propose a prompt-based contrastive learning approach for unsupervised sentence representation learning. The main contributions are:

- Introduces PSCSE, the first model that merges the idea of negative sentence construction using a prompt and the filtering of false negatives to achieve the goal of a uniform representation of the sentence spaces.
- Enlarges the negative sample space through semantic perturbation brought about by the prompt, thus reducing the semantic bias caused by the common random negative sampling approach.
- Proposes a semantic-similarity-based Sample Filter using a dedicated auxiliary encoder that is unsupervised and aims to remove the false negatives that could cause similar semantic statements to be pushed apart.
- Brings considerable gains to the unsupervised STS task, beating the current best baselines SimCSE, E-SimCSE, and PromptBERT, with a maximum gain of 1.0 on average Spearman’s correlation.
- Offers a flexible and integrable sentence-level encoder that could be used for downstream semantic retrieval, question answering, and dialog modeling. Recognize first and foremost that our existing assessment revolves around ensuring STS benchmarks. However, as was mentioned in context, in following works, PSCSE will be tested on other formal tasks aside from embedding quality in unsupervised settings, such as in dense retrieval and in question answering.
- Improves semantic consistency and diminishes semantic confusion that might occur during the interaction between human and computer during NLP.
- Ultimately, with its widespread impacts on society, i.e., reducing costs in developing strong models in Natural Language Processing by our efficient data building paradigm and thus helping research teams with fewer resources and developing specific applications for particular groups, i.e., helping in developing low-resource language models and health care management systems, a more inclusionary path in AI will follow.

## Data Availability

No new datasets were generated or collected in the current study. The source code for data processing, model training, and evaluation is publicly available at: https://github.com/l6235/PSCSE.

## Footnotes

1 https://huggingface.co.
